# Recent Strategies for Lithium-Ion Conductivity Improvement in Li_7_La_3_Zr_2_O_12_ Solid Electrolytes

**DOI:** 10.3390/ijms241612905

**Published:** 2023-08-17

**Authors:** Evgeniya Il’ina

**Affiliations:** Laboratory of Electrochemical Power Sources, Institute of High Temperature Electrochemistry, Ural Branch of the Russian Academy of Sciences, Yekaterinburg 620990, Russia; ilyina@ihte.uran.ru

**Keywords:** Li_7_La_3_Zr_2_O_12_, garnet structure, doping, lithium-ion conductivity, all-solid-state batteries

## Abstract

The development of solid electrolytes with high conductivity is one of the key factors in the creation of new power-generation sources. Lithium-ion solid electrolytes based on Li_7_La_3_Zr_2_O_12_ (LLZ) with a garnet structure are in great demand for all-solid-state battery production. Li_7_La_3_Zr_2_O_12_ has two structural modifications: tetragonal (*I41/acd*) and cubic (*Ia3d*). A doping strategy is proposed for the stabilization of highly conductive cubic Li_7_La_3_Zr_2_O_12_. The structure features, density, and microstructure of the ceramic membrane are caused by the doping strategy and synthesis method of the solid electrolyte. The influence of different dopants on the stabilization of the cubic phase and conductivity improvement of solid electrolytes based on Li_7_La_3_Zr_2_O_12_ is discussed in the presented review. For mono-doping, the highest values of lithium-ion conductivity (~10^−3^ S/cm at room temperature) are achieved for solid electrolytes with the partial substitution of Li^+^ by Ga^3+^, and Zr^4+^ by Te^6+^. Moreover, the positive effect of double elements doping on the Zr site in Li_7_La_3_Zr_2_O_12_ is established. There is an increase in the popularity of dual- and multi-doping on several Li_7_La_3_Zr_2_O_12_ sublattices. Such a strategy leads not only to lithium-ion conductivity improvement but also to the reduction of annealing temperature and the amount of some high-cost dopant. Al and Ga proved to be effective co-doping elements for the simultaneous substitution in Li/Zr and Li/La sublattices of Li_7_La_3_Zr_2_O_12_ for improving the lithium-ion conductivity of solid electrolytes.

## 1. Introduction

Lithium-ion batteries are widely used in many aspects of human life. The high energy density and long cycle life are the main advantages of such power sources [1]. However, according to the limited theoretical energy density of conventional lithium-ion batteries, the possibility of liquid electrolyte leakage, and the subsequent flammability of lithium-ion batteries, the interest in alternative power sources has grown. For example, lithium-air batteries are considered to be promising candidates for next-generation rechargeable batteries because of their high theoretical energy density [2]. Aqueous rechargeable lithium batteries, based on low-cost and environmentally friendly water electrolytes [3,4,5], are another power source. A significant increase in the number of publications in the global scientific literature [6,7,8,9,10], as well as patents on all-solid-state batteries (ASSB), indicates active scientific and design work in this direction. ASSB can work in broader conditions (elevated temperatures, high pressures, and aggressive atmospheres) than traditional lithium-ion power sources with liquid electrolytes [6,7,8,9,10]. The compounds with different structures considered to be lithium-ion solid electrolytes for ASSB are perovskite-type, LISICON-type, NASICON-type, and garnet-like structured solid conductors [11,12,13,14,15,16,17,18,19,20,21]. Solid electrolytes based on Li_7_La_3_Zr_2_O_12_ (LLZ) with a garnet structure are in great demand. The uniqueness of this lithium-conducting solid electrolyte is the combination of high lithium-ion conductivity (~10^−4^ S/cm at 25 °C) with stability versus lithium. Li metal is characterized by high values of theoretical capacity (3860 mAh/g) and low density (0.59 g/cm^3^) [22], so high-energy power sources can be developed based on the LLZ solid electrolyte and Li anode.

Orthosilicates with the A_3_^II^B_2_^III^(SiO_4_) (A = Ca, Mg, Fe; B = Al, Cr, Fe, where A and B are 8- and 6-fold coordinated by oxygen cations, respectively) general formula form a large family of minerals, which is related to the structural type of the garnet. A, B, and Si sites can be occupied by cations of different natures and oxidation states (alkaline, alkaline earth, rare earth, and transition metals) in the garnet structure. The known nonsilicate structures of garnets have the A_3_M_2_(MO_4_)_3_ (A = Y, Gd; M = Fe, Al, Ga) formula and crystallize in the space group *Ia3d* [23]. Lithium-ion conductors with the garnet structure were obtained by Hayashi and Noguchi in 1986 [24,25]. They reported new phases of La_3_Li_7_Ta_2_O_13_ and La_3_Li_7_Nb_2_O_13_ with cubic structure. Then, the composition was refined and Mazza et al. [26] noted that La_3_Li_5_M_2_O_I2_ (M = Ta, Nb) lithium garnets are unique compounds because they contain a large trivalent cation—La^3+^. In addition, lithium ions occupy octahedral voids centered at ¼, ¼, ¼ inside the unit cell, which are usually free in garnets.

However, Trangadurai et al. [27] turned their attention to La_3_Li_5_M_2_O_I2_ (M = Ta, Nb) garnets as the new highly conductive lithium-ion conductors with structural disorder in only 2003. They suggested that these phases should have high lithium-ion conductivity since they have many free lithium positions in the structure. The bulk conductivity for both garnets was equal to ~10^−6^ S/cm at 25 °C. Moreover, it was found that Li_5_La_3_Ta_2_O_12_ is stable in contact with molten lithium, and it is characterized by a high decomposition voltage (6 V relative to Li^0^/Li^+^) [27]. Therefore, the Li_5_La_3_Ta_2_O_12_ solid electrolyte can be considered to be a promising material for lithium power sources. However, the total conductivity of ceramics had to be improved. Therefore, in later years, active work was carried out to expand several compounds with the garnet-like structure using chemical substitutions and structural modifications [20]. In 2007, the group of Murugan [28] synthesized one of the most highly conductive compounds among lithium-ion conductors with a garnet structure—Li_7_La_3_Zr_2_O_12_ (~10^−4^ S/cm at room temperature). Then, it was established that Li_7_La_3_Zr_2_O_12_ has two structural modifications: cubic (*Ia3d*) and tetragonal (*I41/acd*) [20]. Tetragonal modification possesses lower conductivity values, namely ~10^−6^ S/cm at room temperature. A cubic modification with high conductivity can be obtained only by the introduction of some dopant in the LLZ structure [20]. Therefore, the active study of different doping elements on chemical structure, phase composition, and lithium-ion conductivity of LLZ has been ongoing since 2011.

It should be noted that the doping of Li_7_La_3_Zr_2_O_12_ can not only lead to lithium-ion conductivity growth but also to the densification of ceramic and its microstructure modification. The high-density ceramic membrane, in turn, prevents the formation of lithium dendrites, which can be observed in lithium power sources during electrochemical testing [20,29].

The effect of structural substitutions in the different sublattices of Li_7_La_3_Zr_2_O_12_ compounds, including mono-, dual-, and multi-doping, on cubic phase stabilization and the conductivity of solid electrolytes based on LLZ has been studied in the present review.

## 2. Results and Discussions

### 2.1. Structural Features of the Tetragonal and Cubic Modifications of Li_7_La_3_Zr_2_O_12_

**Figure 1:**
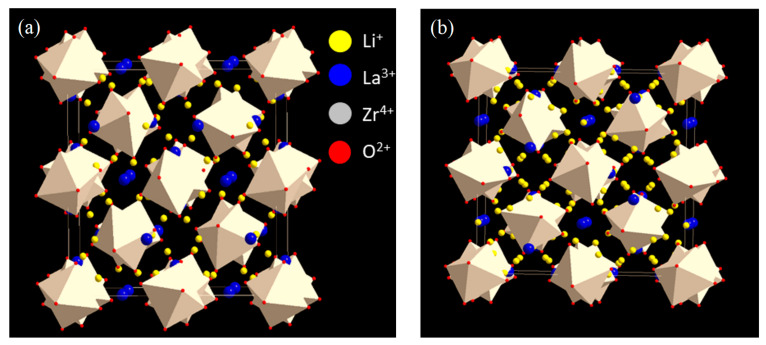
Cubic (**a**) and tetragonal (**b**) structures of Li_7_La_3_Zr_2_O_12_.

Li_7_La_3_Zr_2_O_12_ obtained by Murugan et al. [28] had cubic modification with the lattice parameter a = 129,682(6) Å. The stabilization of the cubic structure was achieved by partial penetration of Al from an Al_2_O_3_ crucible under high-temperature sintering. In the cubic structure of LLZ (Figure 1a), La, Zr, and O atoms occupy 24c, 16a, and 96h sites, respectively [30]. According to Awaka et al. [30], the garnet framework structure was composed of dodecahedral LaO_8_ and octahedral ZrO_6_. Li atoms (Li1 and Li2) occupied two types of crystallographic sites in the framework of LLZ: tetrahedral 24d and distorted octahedral 96h. The occupancy of the tetrahedral site in LLZ (g = 0.94) is the highest among all known lithium compounds, with the garnet structure. The octahedral site is only partially occupied by lithium ions (g = 0.349). The very short distance between lithium atoms along the migration path is an important feature of the LLZ cubic modification, which also leads to high conductivity. According to Geiger et al. [31], the high conductivity of cubic LLZ is caused by several structural features: the Li^+^ partial occupation of the tetrahedral 24 d site, the short distances between Li sites for lithium-ion migration, and the static disorder of Li atoms in the doped cubic structure of LLZ.

The presence of the tetragonal modification of LLZ was established in 2009 [32], although most lithium-conducting garnet complex oxides had a cubic structure with the space group *Ia3d*. According to the crystallographic data obtained on the LLZ single crystal, the compound had a tetragonal structure with the space group *I41/acd* and lattice parameters a = 13.134(4) Å and c = 12.663(8) Å (Figure 1b). The structure of tetragonal LLZ consists of La(1)O_8_ and La(2)O_8_ polyhedra and ZrO_6_ octahedra. Lithium atoms occupy three types of crystallographic sites: tetrahedral site 8a, and distorted octahedral sites 16f and 32g. Lanthanum atoms occupy two types of crystallographic sites: 8b and 16e, while Zr and O atoms occupy 16c and 32g sites, respectively. An important difference between the two modifications of LLZ is that in a tetragonal structure, lithium atoms are characterized by the complete occupancy of all three available sites, which leads to lower values of its bulk conductivity (1.6 × 10^−6^ S/cm at 27 °C).

**Figure 2:**
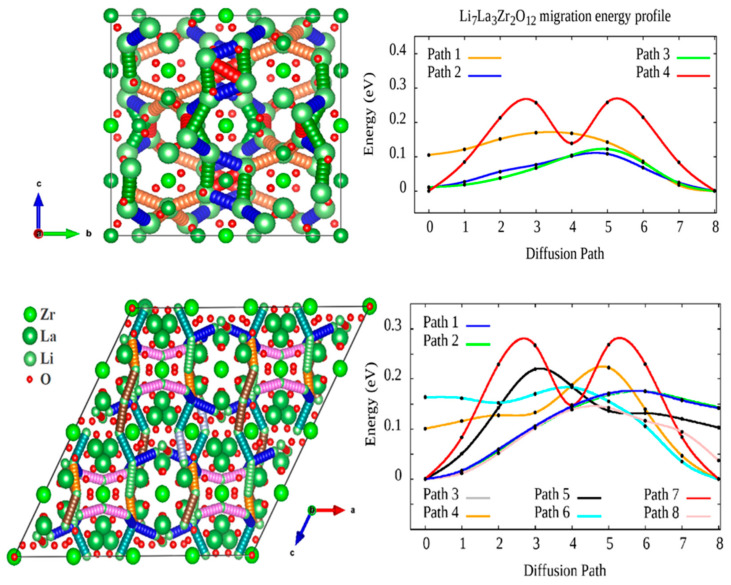
Scheme of Li diffusion channels in the tetragonal and cubic LLZ structures and the corresponding migration energy barriers for each channel according to the DFT-NEB methodology [33,34]. Reprinted with permission from [34]. Copyright 2022, American Chemical Society.

Pair distribution function (PDF) analysis was performed for tetragonal and cubic LLZ in our previous work [33,34], Figure 2. The migration energies of Li^+^ ions in both structures were calculated using the Density Functional Theory (DFT) approach using the Nudged Elastic Band (NEB) method. It was established that tetragonal LLZ possesses a 3-periodic diffusion map, which is formed by four nonequivalent paths, while the cubic structure of LLZ has eight nonequivalent paths for Li^+^ ion diffusion, which forms the 3D migration map.

The doping mechanism is used for cubic phase stabilization of Li_7_La_3_Zr_2_O_12_. The introduction of various dopants leads to structural changes in the crystal lattice of the initial compound, which in turn have a significant influence on the bulk conductivity of solid electrolytes. Thompson et al. [35] proposed that the electrostatic repulsion of the Li^+^–Li^+^ pair or Li^+^–dopant ion pair leads to the Li rearrangement in LLZ structure between the tetrahedral and octahedral sites, and defines the effective carrier concentration.

### 2.2. Structural Substitutions in Different Sublattices of the Li_7_La_3_Zr_2_O_12_ Compound

#### 2.2.1. Li Sublattice

The partial substitution of Li^+^ by divalent and trivalent ions in the LLZ structure is carried out to create additional lithium vacancies, which leads to cubic phase stabilization. Aluminum is the most commonly used dopant for lithium substitution because doping by this element is carried out using an Al-containing precursor or by the uncontrolled introduction of aluminum from the alundum crucible during the high-temperature annealing of ceramics. Lithium-ion conductivity values of Al-doped solid electrolytes are equal to ~10^−4^ S/cm at 25 °C [20,28]. Li_7−3x_Al_x_La_3_Zr_2_O_12_ solid electrolytes were obtained by various methods (solid-state reaction, sol–gel, spark plasma sintering, self-consolidation method) and under different final heat-treatment conditions, which have a significant effect on the lithium content in the compound, the stabilization of highly conductive cubic modification, the phase composition and density of ceramic membranes, and, as a result, on the total conductivity of the obtained solid electrolytes [20,36,37,38,39,40,41,42,43,44,45,46,47]. Rangasamy et al. [36] studied the effect of the Li and Al ions concentration on the cubic LLZ formation. It was established that the introduction of 0.204 mol Al leads to the stabilization of cubic modification. Then, the influence of Li^+^ concentration in Al-doped LLZ on the phase composition and phase transition was investigated. It was found that the impurity phase in the form of lanthanum zirconate appeared during the low lithium content in Li_8–x_Li_0.48_La_3_Zr_2_Al_0.24_O_12.62_, while lithium excess led to the formation of tetragonal LLZ. According to the obtained results, it was concluded that the concentrations of dopant element and Li play key roles in the cubic phase formation. Furthermore, Zhuang et al. [44], using the Distribution of Relaxation Times (DRT) method, established that the low conductivity of Li_7−3x_Al_x_La_3_Zr_2_O_12_(x = 0.00–0.40) solid electrolytes with low Al content is caused by the residual tetragonal phase, while the low conductivity of ceramics with high Al content is caused by the segregation of the impurity phases (LiAlO_2_ and LaAlO_3_). According to the molecular dynamic simulation and advanced atomistic simulation technique, it was shown that ideal upper and lower limits for Al concentrations are between 0 and 0.25 Al per formula unit (pfu) [45].

Duvel et al. [46] established that the observed high ionic conductivity (10^−4^ S/cm at room temperature) of Al-doped LLZ depends not only on the stoichiometry and the selected annealing conditions, but also on the exact distribution of Al over various sites in the Li_7_La_3_Zr_2_O_12_ structure. To study the location of aluminum in the LLZ structure, Li_7−3x+z_Al_x+y+z_La_3−y_Zr_2−z_O_12_ solid electrolytes were obtained using mechano synthesis, which helps to control the cation ratio. At low Al concentrations, Al^3+^ ions act as an alivalent impurity and replace three Li^+^ ions. However, the La^3+^ and Zr^4+^ ions are gradually replaced by aluminum ions with Al content increasing. The substitution of Al^3+^ by La^3+^ and Zr^4+^ leads to the stabilization of cubic modification and significantly affects the Li^+^ dynamic in the garnet structure. 

**Figure 3:**
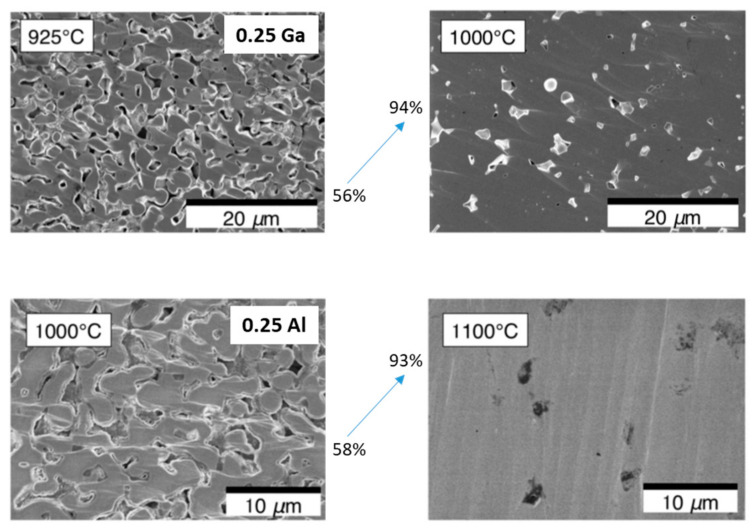
Cross-sectional SEM micrographs of Li_6.25_Ga_0.25_La_3_Zr_2_O_12_ and Li_6.25_Al_0.25_La_3_Zr_2_O_12_ solid electrolytes sintered at various temperatures [47]. Reprinted from [47], with permission from Elsevier B.V.

Ga^3+^ is another successfully used doping element [20,47,48,49,50,51,52,53]. The effect of Ga-doping on the structure of garnet-type LLZ was investigated using force-field-based simulation [49]. Similar to Al^3+^, the ionic radius of Ga^3+^ (0.47 Å and 0.62 Å for tetrahedral and octahedral coordination, respectively) is also comparable to the Li^+^ ionic radius. It was shown that Ga^3+^ incorporation does not change the lattice constant and does not contribute to any significant structural distortion. Jalem et al. [50] investigated the Li_6.25_La_3_Zr_2_Ga_0.25_O_12_ composition because LLZ cubic phase was earlier stabilized by the introduction of 0.25 mol of aluminum. The solid electrolytes obtained by hot pressing had cubic structure and high values of total conductivity, namely 3.5 × 10^−4^ S/cm at room temperature. Matsuda et al. [47] also chose the Li_6.25_La_3_Zr_2_Ga_0.25_O_12_ composition and studied the sintering features of these ceramics. The optimal heat-treatment conditions were chosen, under which the highest values of total conductivity were achieved—9.6 × 10^−4^ S/cm for Ga-doped ceramic (1000 °C for 12 h), and 4.5 × 10^−4^ S/cm for Al-doped LLZ (1100 °C for 12 h). These elements act as sintering additives; their addition leads to the significant growth of the samples’ relative densities with the annealing temperature increasing; see Figure 3. Sharifi et al. [52] synthesized Li_7−3x_Ga_x_La_3_Zr_2_O_12_ (x = 0.2–0.3) solid electrolytes with cubic structure using the sol–gel method. The solid electrolyte with x = 0.2 exhibited the highest conductivity of 5.85 mS/cm at 20 °C. Han et al. [53] showed that Li_7−3x_Ga_x_La_3_Zr_2_O_12_ solid electrolytes, as well as high conductivity values, exhibited the highest fracture stress (∼143 MPa) and fracture toughness in comparison with Al- and Ta-doped LLZ. Al- and Ga-doped LLZ solid electrolytes are stabile to metallic lithium [20].

Moreover, the influence of other doping elements introduced in the lithium sublattice on the structure, phase composition, and conductivity of the LLZ solid electrolyte was studied [54,55,56,57,58,59]. Brugge et al. [54] synthesized Li_7−4x_Ge_x_La_3_Zr_2_O_12_ (x = 0.05–0.50) solid electrolytes using the sol–gel method. The cubic phase was stabilized by the introduction of 0.10 mol Ge; this composition had the highest value of bulk conductivity—2.8 × 10^−4^ S/cm at 25 °C. Amardeep et al. [55] synthesized Mg-doped solid electrolytes using the sol–gel method with cubic structure and lithium-ion conductivity of 1.1 × 10^−4^ S/cm. Jiang et al. [56] stabilized the cubic modification of LLZ by the partial substitution of Li^+^ by Zn^2+^. The samples were obtained by solid-state synthesis method and had low conductivity values—4.5 × 10^−5^ S/cm at 20 °C (x = 0.025). Moreover, it should be noted that, according to the elemental analysis, sintered samples contain 0.25 mol Al.

In work by Xiang et al. [57], Li_6.4_A_x_La_3_Zr_2_O_12_ (A = Be, B, Al, Fe, Zn, Ga, x = 0.2/0.3) solid electrolytes were prepared using the solid-state method. It was established that the partial substitution of Li^+^ by Be^2+^, B^3+^, or Zn^2+^ even up to 0.5 did not lead to the stabilization of cubic modification. Therefore, it can be assumed that the cubic phase of LLZ in work [56] was stabilized by the introduction of Al. Compared with Al-doped LLZ, the conductivity of Fe- or Ga-doped LLZ shows a greater improvement. The solid electrolyte with gallium addition had the highest conductivity values among Li_6.4_A_0.2_La_3_Zr_2_O_12_ (A = Al, Fe, Ga) solid electrolytes—1.31 × 10^−3^ S/cm at room temperature. The room-temperature total conductivities of solid electrolytes with aluminum and iron were equal to 3.3 × 10^−4^ and 8.2 × 10^−4^ S/cm, respectively. The effect of iron doping was also studied [58,59]. Rettenwander et al. [58] synthesized Li_7−3x_Fe_3+x_La_3_Zr_2_O_12_ solid electrolyte with x = 0.19 using the solid-state method. Mössbauer spectra show that about 96% of the iron was Fe^3+^ and only 4% was Fe^2+^. Approximately two thirds of the Fe^3+^ cations occupy the tetrahedral site (24d); about one quarter is in the highly distorted site (possibly at 96h) of the garnet structure, and the remaining iron ions occupy other crystallographic sites. Li_7−3x_Fe_x_La_3_Zr_2_O_12_ (Fe^3+^) garnets with x = 0.04–0.72 pfu were synthesized using the high-temperature solid-state method [59]. The cubic modification was successfully stabilized by the addition of 0.16 mol Fe. Mössbauer measurements show that about 80% of the Fe^3+^ ions were located at the 24d site, and about 20% at the general 96h site.

**Figure 4:**
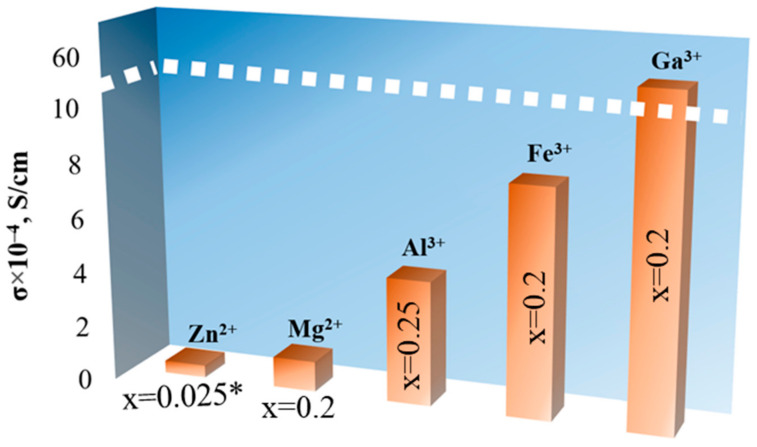
Total conductivity of solid electrolytes with the partial substitution of Li^+^ in Li_7_La_3_Zr_2_O_12_ structure. * 0.25 mol Al [56].

The partial substitution of Li^+^ by alivalent ions (Mg^2+^, Al^3+^, Fe^3+^, Ga^3+^, Ge^4+^) in the LLZ structure leads to cubic phase stabilization, which is caused by the creation of additional lithium vacancies and Li rearrangement between tetrahedral and octahedral sites. It can be concluded that the introduction of 0.2–0.25 mol dopant is required for LLZ stabilization and the achievement of high conductivity values; see Figure 4. The relative densities of the ceramic samples presented in Figure 4 were 91–95%. The partial substitution of Li^+^ by Ga^3+^ in the LLZ structure is the most optimal strategy for cubic modification stabilization and Li^+^ conductivity improvement. This element (as well as Al) acts as a sintering additive and improves not only the bulk but also the grain-boundary conductivity of the ceramic membranes. The gallium-doped solid electrolyte with a record value of lithium-ion conductivity (5.85 mS/cm at 20 °C) was obtained due to the replacement of the traditional solid-phase synthesis method with the sol–gel method [52].

#### 2.2.2. La Sublattice

**Figure 5:**
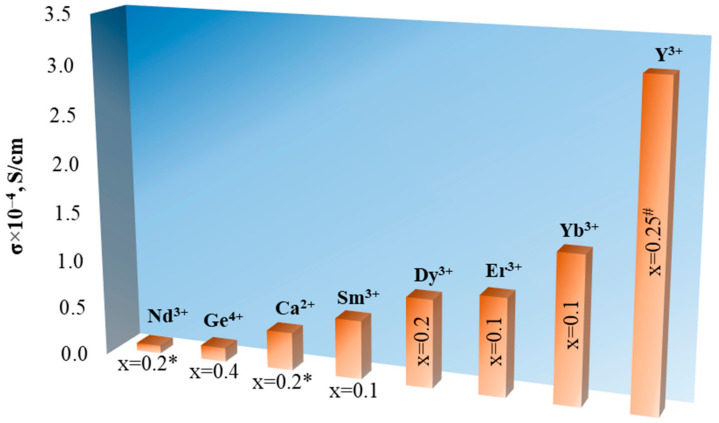
Total conductivity of solid electrolytes with the partial substitution of La^3+^ in Li_7_La_3_Zr_2_O_12_ structure. * 0.1–0.4 mol Al [60]; ^#^ 0.64 wt% Al [61].

The substitution of some lanthanum ions for cubic phase stabilization is the less common method of LLZ doping. The results of LLZ doping with divalent, trivalent, and tetravalent ions over the sublattice of trivalent lanthanum are presented in the literature [60,61,62,63,64]; see Figure 5.

Hanc et al. [60] studied the effect of the partial substitution of lanthanum ions by divalent calcium and trivalent neodymium. It was established that the introduction of dopants with a minimum content (x = 0.2) leads to cubic phase stabilization. At the same time, the transition from tetragonal to cubic structure was observed only after the heat treatment of the samples at 1200 °C. Such sintering conditions can lead to the partial volatilization of lithium (the lithium content ranged from 5.79 to 6.12 for the obtained solid electrolytes) and/or additional introduction of aluminum from the crucible (LaAlO_3_ impurity was observed). The introduction of Nd dopant led to the decrease of lattice parameters and room-temperature lithium-ion conductivity (4.2 10^−5^ and 8.1 × 10^−6^ S/cm for undoped LLZ and Li_7_La_2_NdZr_2_O_12_, respectively). Ca-doped LLZ had higher values of total conductivity, ~3.9 × 10^−5^ S/cm at room temperature.

Deviannapoorani et al. [61] used Y^3+^ for the partial substitution of La^3+^ in the LLZ structure. It was established that the introduction of the yttrium dopant (x = 0.125) led to cubic phase stabilization. Li_7_La_2.75_Y_0.25_Zr_2_O_12_ solid electrolyte has high density and maximum value of lithium-ion conductivity—3.21 × 10^−4^ S/cm at 30 °C. Such high lithium-ion conductivity values of Y-doped LLZ are caused by the fact that this dopant element also acts as a sintering aid, which leads to sample densification and, as a result, total conductivity growth. Park et al. [62] synthesized Li_7_La_3−x_Zr_2_O_12_—xEu^3+^ (0.02 ≤ x ≤ 0.10) solid electrolytes using the solid-state method. Stabilization of the cubic phase has not been achieved; the samples were a mixture of tetragonal and cubic modifications. Only the photoluminescence properties of the obtained compounds were studied. Abdulai et al. [63] stabilized the cubic structure of LLZ by A^2+^ (Sm, Dy, Er, Yb) doping at La^3+^ sites. It was found that the solubility limit of lanthanide group cations reduced with a decrease in the ionic radius of the introduced element. The cubic phase stabilization was observed after the dopant introduction of x = 0.1. However, according to X-ray diffraction data, the initial composition (without doping) also had a cubic modification. This can be explained by the additional introduction of aluminum into the ceramics due to the heat treatment of the solid electrolyte in alundum crucibles. The highest ionic conductivity was achieved for Yb-doped LLZ—1.5 × 10^−4^ S/cm at room temperature.

Rangasamy et al. [64] obtained Li_7−x_La_3−x_Ce_x_Zr_2_O_12_ (x = 0–0.8) solid electrolytes with the partial substitution La^3+^ by Ce^4+^ using the solid-state synthesis method. It was established that the introduction of x = 0.4 led to cubic modification stabilization. However, all obtained solid electrolytes contained impurities in the form of cerium oxide (CeO_2_). The total conductivity of Li_6.6_La_1.6_Ce_0.4_Zr_2_O_12_ was equal to 1.44 × 10^−5^ S/cm at room temperature.

The maximum values of room-temperature total conductivity of the solid electrolytes with the partial substitution of La^3+^ in the Li_7_La_3_Zr_2_O_12_ structure are shown in Figure 5. These solid electrolytes were obtained by solid-state synthesis, except for Y-doped LLZ (sol–gel method). It can be seen that solid electrolytes based on LLZ with the partial substitution of La^3+^ by Y^3+^ have the highest conductivity values; such conductivity improvement of initial LLZ can be also caused by the Al introduction (0.64 wt% Al) and use of sol–gel synthesis. The total conductivity of solid electrolytes with the partial substitution of La^3+^ in Li_7_La_3_Zr_2_O_12_ is lower than for Li^+^-substituted garnets.

#### 2.2.3. Zr Sublattice

The partial substitution of Zr^4+^ by two-, three-, four-, five-, and six-valent ions can stabilize the cubic structure of LLZ. Such an introduction leads to the geometry modification of the lattice spacing and the improvement of lithium-ion stoichiometry and conduction channels. Partial substitution of Zr^4+^ by elements with higher valence in the LLZ structure is carried out to create additional lithium vacancies. 

##### A^2+^ Doping

The possibility of cubic structure stabilization by A^2+^ doping on Zr^4+^ sites has been shown [56]. Jiang et al. [56] used the partial substitution of zirconium by magnesium; ceramics were synthesized using the solid-state method. The solid electrolyte with x = 0.1 addition of magnesium had the highest conductivity values—2.91 × 10^−4^ S/cm at 20 °C. It should be noted that the obtained samples contained Al (mainly along the grain boundaries of LLZ), which can also lead to cubic phase stabilization and conductivity improvement of the obtained samples. 

##### A^3+^ Doping

The cubic structure of Li_7_La_3_Zr_2_O_12_ can be also stabilized by A^3+^ doping on Zr^4+^ sites. It was supposed that excess Li^+^ occupies the octahedral sites in the LLZ structure and leads to an increase in ionic conductivity due to Li^+^ carrier growth. Song et al. [65] obtained a Li_7.1_La_3_Zr_1.9_Cr_0.1_O_12_ solid electrolyte using the solid-state method with lithium-ion conductivity of 5.2 × 10^−4^ S/cm at 27 °C. Sintering was carried out at 1230 °C for 16 h, so such high conductivity values and cubic phase stabilization may be caused by the partial incorporation of aluminum. Wang et al. [66] used the partial substitution of Zr^4+^ by Sm^3+^; solid electrolytes were synthesized using the solid-state method. It was found that the optimal addition of Sm^3+^ in Li_7+x_La_3_Zr_2−x_Sm_x_O_12_ is equal to 0.06 (2.46 × 10^−4^ S/cm at 20 °C). The obtained solid electrolyte can contain Al because undoped LLZ also had a cubic structure. Song et al. [67] synthesized Li_7+x_La_3_Zr_2−x_Gd_x_O_12_ (x = 0.1–0.5) solid electrolytes with a cubic structure using the solid-state method. The highest conductivity was achieved for ceramic with x = 0.2–2.3 × 10^−4^ S/cm at room temperature. Moreover, the obtained solid electrolyte demonstrates chemical stability in contact with Li at room temperature. Jiang et al. [56] used partial substitution of zirconium by scandium. The conductivity value of Sc-doped LLZ with x = 0.05 was equal to 1.65 × 10^−4^ S/cm at 20 °C. Murugan et al. [68] used Y^3+^ (3% Y stabilized ZrO_2_) for partial substitution of Zr^4+^ ions. Li_7.06_La_3_Y_0.06_Zr_1.94_O_12_ ceramic was obtained using the solid-state method and had the cubic structure and total conductivity of 8.1 × 10^−4^ S/cm at 25 °C. However, the obtained samples contained 1.6 wt.% Al from Al_2_O_3_ crucibles. That is why Kotobuki et al. [69] obtained Al-free Y-doped Li_7_La_3_Zr_2_O_12_ of the same composition using a spark plasma sintering technique. The increase of sintering temperature from 800 to 1100 °C leads to the Li content decrease from 6.94 to 6.43, respectively. The solid electrolyte sintered at 1100 °C had a maximum conductivity value—9.8 × 10^−4^ S/cm at 25 °C.

##### A^4+^ Doping

The stabilization of the LLZ cubic structure was carried out by A^4+^ doping on Zr^4+^ sites in works [70,71]. Hu et al. [70] used Ge^4+^ as a doping element, which occupies Zr^4+^ sites in the garnet structure. It was established that the introduction of 0.1 mol Ge led to the cubic modification stabilization. Li_7_La_3_Zr_1.7_Ge_0.3_O_12_ synthesized by solid-state method exhibited the highest total conductivity of 4.78 × 10^−4^ S/cm at 20 °C. Wang et al. [71] used Ti^4+^ for the partial substitution of Zr^4+^ ions. Solid electrolytes were synthesized at low temperatures (1120 °C) by chemical co-precipitation. Ti doping led to ionic conductivity growth of up to 1.94 × 10^−4^ S/cm at 25 °C for Li_7_La_3_Zr_1.9_Ti_0.1_O_12_.

##### A^5+^ Doping

The pentavalent elements tantalum and niobium are the most commonly used dopants for Zr^4+^ substitution in the LLZ structure. Such doping leads to the formation of additional lithium vacancies, increasing the disorderliness in the garnet structure, and cubic phase stabilization. Solid electrolytes in Li_7−x_La_3_Zr_2−x_Ta_x_O_12_ and Li_7−x_La_3_Zr_2−x_Nb_x_O_12_ systems have been obtained by different methods (solid-state and sol–gel synthesis) and under various treatment conditions, which is why the maximum conductivity was established for compounds with different Ta and Nb content, from x = 0.25 to x = 1.0 [20,34,35,72,73,74,75,76,77,78,79,80,81]. Moreover, the uncontrolled introduction of aluminum from crucibles or the addition of some Al_2_O_3_ was observed in some works [73,80,81], which leads to the improvement of lithium-ion conductivity in Ta- or Nb-doped ceramics. 

Ohta et al. [73] synthesized Li_7−x_La_3_Zr_2−x_Nb_x_O_12_ (x = 0–2) membranes with the cubic structure using the solid-state method and suggested that the increase in conductivity is associated with the mobility growth of charge carriers. The compound with x = 0.25 had the highest value of total conductivity—8.0 × 10^−4^ S/cm at 25 °C. In our previous work [74], Li_7−x_La_3_Zr_2−x_Nb_x_O_12_ solid electrolytes were synthesized using the sol–gel method, and the stabilization of cubic modification occurred at x > 0.1. Solid electrolytes with x = 0.25 also exhibited the maximum value of total conductivity—4.0 × 10^−5^ S/cm at room temperature. Such a high difference in conductivity can be explained by the partial introduction of Al in the solid electrolyte structure [73]. For the improvement of Li-ion conductivity of Al-free Li_7−x_La_3_Zr_2−x_Nb_x_O_12_ ceramics, Zhao et al. [75] used a self-consolidation strategy, and Reis et al. [76] used a spark plasma sintering technique at 950 °C for 10 min. The maximum total conductivity was achieved for compositions with x = 0.6 (5.22 × 10^−4^ S/cm at 30 °C) [75] and x = 1.0 (1.9 × 10^−4^ S/cm at 25 °C) [76].

Al-free Li_7−x_La_3_Zr_2−x_Ta_x_O_12_ solid electrolytes were synthesized in works [34,77,78,79]; as mentioned above, the maximum conductivity was observed for different compositions. For example, Inada et al. [77] used solid-state synthesis and Li_6.5_La_3_Zr_1.5_Ta_0.5_O_12_ solid electrolyte had the maximum conductivity—6.1 × 10^−4^ S/cm. In Ref. [78], solid electrolytes with x = 0.5 also possessed the highest lithium-ion conductivity—3.54 × 10^−4^ S/cm. Wang et al. [79] synthesized Li_6.7_La_3_Zr_1.7_Ta_0.3_O_12_ using the solid-state method with the maximum value of room-temperature conductivity—4.78 × 10^−4^ S/cm. Thompson et al. [35] used hot pressing to obtain dense samples of Li_7−x_La_3_Zr_2−x_Ta_x_O_12_ solid electrolytes, which led to the formation of a highly conductive ceramic membrane (8.16 × 10^−4^ S/cm at room temperature for Li_6.5_La_3_Zr_1.5_Ta_0.5_O_12_ solid electrolyte). The samples obtained using the hot-pressing technique (without sintering aid) have high density, similar grain sizes, and microstructures. In our previous work [34], the solid electrolyte with x = 0.6 synthesized by sol–gel method had the maximum value of conductivity (1.4 × 10^−4^ S/cm at 25 °C). Using density functional theory modeling, it was confirmed that the doping of Ta^5+^ ions up to x < 1.0 is most suitable for Li^+^ diffusion enhancing and Li-ion conductivity improvement in solid electrolytes based on LLZ.

Bi and Sb were also used for A^5+^ doping on Zr^4+^ sites in LLZ structure [82,83,84]. Schwanz et al. [82] synthesized Li_7−x_La_3_Zr_2−x_Bi_x_O_12_ solid electrolytes using the sol–gel Pechini method; a solid electrolyte with x = 0.75 had the highest value of lithium-ion conductivity—2.0 × 10^−4^ S/cm at 27 °C. Ramakumar et al. [83] obtained an Sb-doped LLZ with x = 0.2–1.0 using the solid-state method. The maximum conductivity was achieved for single-phase composition with x = 0.4–7.7 × 10^−4^ S/cm at 30 °C. It was noted that Sb content growth leads to an increase in the lithium-ion number in tetrahedral sites with a corresponding decrease in the octahedral site occupancy. The optimal concentration of Li^+^ in tetrahedral and octahedral sites was achieved for the composition with x = 0.4. Liang et al. [84] obtained an Sb-doped LLZ (solid-state method) with lower values of total conductivity—9.7 × 10^−5^ S/cm at 30 °C (x = 0.3). Such difference in the conductivity of Sb-doped LLZ may be caused by different Al content in solid electrolytes due to its possible introduction from the crucible (undoped LLZ had the cubic structure [83,84]).

##### A^6+^ Doping

The hexad-substituted LLZ solid electrolytes were obtained [85,86,87,88] using the solid-state method. Li_7−2x_La_3_Zr_2−x_M_x_O_12_ (Mo, Cr, and W) solid electrolytes were studied by Gao et al. [85]. The obtained solid electrolytes had a cubic structure, and W-substituted ceramic with x = 0.2 had the highest total conductivity of 8.7 × 10^−5^ S/cm. Li et al. [86] also stabilized the cubic structure of LLZ by the partial substitution of Zr^4+^ by W^6+^ (x = 0.15–0.55). The obtained solid electrolyte had higher values of total conductivity, 6.6 × 10^−4^ S/cm at 25 °C (Li_6.3_La_3_Zr_1.65_W_0.35_O_12_). Rettenwander et al. [87] used the partial substitution of Zr^4+^ by Mo^6+^ for the cubic phase stabilization of LLZ (x = 0.10–0.40). The bulk conductivity of Li_6.5_La_3_Zr_1.75_Mo_0.25_O_12_ was equal to 3.0 × 10^−4^ S/cm at room temperature. The highest total conductivity values were achieved for Te-doped LLZ by Deviannapoorani et al. [88], 1.02 × 10^−3^ S/cm at 30 °C (Li_6.5_La_3_Zr_1.75_Te_0.25_O_12_). However, it should be noted that this solid electrolyte contains 2.95 wt.% Al.

**Figure 6:**
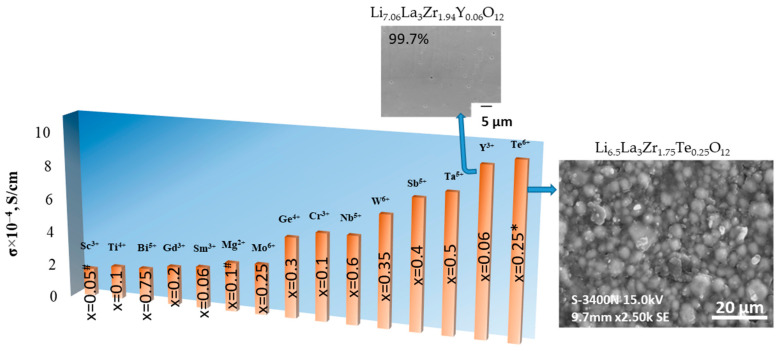
Total conductivity of solid electrolytes with the partial substitution of Zr^4+^ in Li_7_La_3_Zr_2_O_12_ structure. ^#^ 0.2 mol Al [56]; * 2.95 wt% Al [88]. Reprinted from [69] and [88], with permission from Elsevier B.V.

Therefore, the partial substitution of Zr^4+^ in the LLZ structure is represented in the literature by a wide range of elements with valences from 2 to 6. It can be concluded that the introduction of aliovalent dopants with higher valence in comparison with Zr^4+^ leads to an improvement of lithium-ion conductivity except for Y^3+^; see Figure 6. As mentioned above, high conductivity values of yttrium-stabilized LLZ can be caused by not only structural changes but also ceramic densification. The high density (99.7%) of the Y-doped LLZ [69] was achieved using the spark plasma sintering technique. It can be seen from Figure 6 that with an increase in the valence of the introduced dopant, a larger amount of it is required to achieve high values of conductivity in the system. Maximum conductivity values were achieved for the compositions with x < 0.2 and x > 0.25 for ceramics with the partial substitution of Zr^4+^ by A^2+^ or A^3+^ and A^5+^or A^6+^, respectively.

##### Multi-Element Doping

The use of several atoms with different valence states (multi-element doping) leads to different ionic compensation in the LLZ structure. Solid electrolytes in works [89,90,91,92,93,94,95] were synthesized by the traditional solid-state method and had high conductivity values and stability in contact with metal Li. Xu et al. [89] obtained solid electrolytes based on LLZ with Ce^4+^ and Ta^5+^ partial substitution of Zr^4+^. Co-doping of Li_6.4_La_3_Zr_1.4_Ta_0.6_O_12_ compound by Ce^4+^ ions led to Li^+^ migration channels increasing, which facilitates ion transport and leads to the room temperature ionic conductivity growth up to 1.05 × 10^−3^ S/cm. Zhang et al. [90] obtained Li_7−x+y_La_3_Zr_2−x−y_Nb_x_Sm_y_O_12_ solid electrolytes; such co-doping led to the facilitation of the cubic modification formation and Li_6.7_La_3_Zr_1.5_Nb_0.4_Sm_0.1_O_12_ ceramic reached the highest total conductivity, 1.06 × 10^−3^ S/cm at 30 °C. J. Gai et al. [91] used Nb^5+^ and Y^3+^ for the simultaneous substitution of Zr^4+^ ions in the LLZ structure. The obtained Li_7_La_3_ZrNb_0.5_Y_0.5_O_12_ solid electrolyte reached a conductivity of 8.29 × 10^−4^ S/cm at 30 °C and had improved air stability. Luo et al. [92] used Nb and the Gd co-doping of LLZ. Li_7−x+y_La_3_Zr_2−x−y_Nb_x_Gd_y_O_12_ solid electrolytes had the cubic structure and high values of room-temperature lithium-ion conductivity—9.86 × 10^−4^ S/cm (Li_6.5_La_3_Zr_1.3_Nb_0.6_Gd_0.1_O_12_). Tong et al. [93] used two pentavalent elements (Nb and Ta) for the partial substitution of Zr^4+^. The Li_6.4_La_3_Zr_1.4_Ta_0.3_Nb_0.3_O_12_ solid electrolyte possessed the highest value of room-temperature conductivity—6.06 × 10^−4^ S/cm.

Fu and Kuo used more than two elements with different valences for the simultaneous substitution of Zr^4+^ to improve Li^+^-conductivity and structural and air stability of LLZ [94,95]. It was assumed that four equimolar elements (Zr, Nb, Ta, and Hf) with different valences and atomic radii can maximize the Li-ion vacancy/deficiency and disordered Li sublattice, which in turn can lead to high lithium-ion conductivity. Fu et al. [94] designed a novel high-entropy Li-garnet electrolyte—Li_7_La_3_Zr_0.5_Nb_0.5_Ta_0.5_Hf_0.5_O_12_ (samples contain aluminum due to its introduction from the crucible); high-entropy materials contain no fewer than four different cations or anions at one site. The optimal thermal conditions of sintering to obtain high-conductivity ceramics were established, and the dense samples had a total conductivity of 4.67 × 10^−4^ S/cm at room temperature. Kuo et al. [95] used Ta, Nb, Y, and W for the partial substitution of zirconium—Li_6.4_La_3_Zr_0.4_Ta_0.4_Nb_0.4_Y_0.6_W_0.2_O_12_. The obtained solid electrolyte had greater air stability, good electrochemical stability versus Li, and a high conductivity value—1.16 × 10^−4^ S/cm at 25 °C.

According to the analysis of multi-element doping of LLZ on Zr sites, it can be seen that the use of two elements with different valences for Zr^4+^ substitution is a better strategy for Li^+^-conductivity improvement than a higher variety of elements in the Zr site.

### 2.3. Multi-Doping on Several Sublattices in Li_7_La_3_Zr_2_O_12_

Then, a new strategy for lithium-ion conductivity improvement of garnet-type solid electrolytes by the dual-doping and multi-doping methods was proposed. Depending on the used dopant, such substitution enhances the ionic conductivity of the initial compound:

- Structuring of the better lithium-ion conduction framework. The presence of the second dopant element has an impact on the element distribution in Li sites (tetrahedral and octahedral), which leads to the formation of a unique Li local structure in the garnet electrolyte. For example, Al-doped LLZ was co-doped by Ta^5+^ (dual-doping) [96], which leads to providing more open space for Li-ion transport because of Al shifting from the 24d to 96h Li site. In Ref. [97], the conductivity growth was observed with the increased number of doped ions (from 0 to 3) in the Li_7_La_3_Zr_2_O_12_ structure, which is caused by sample densification and the increasing of the Li occupancy at the tetrahedral site (24d), while the Li occupancy at the octahedral site (96h) does not change [97];
- Ceramic densification. Some doping elements act as sintering additives and not only modify bulk conductivity but also significantly reduce grain-boundary resistance. For example, in Ref. [98], the substitution of Zr^4+^ by Ta^5+^/Ba^2+^ was made to stabilize the cubic modification, while the substitution of Li^+^ by Ga^3+^ leads to Li content optimization and increases ceramic sinterability. In turn, high density and good contact between grains is a very important factor for the air stability improvement of solid electrolytes based on LLZ, since reactions of LLZ with air components initially occur at the grain boundaries.

Moreover, in some cases, a multi-doping strategy leads to reducing the annealing temperature of the ceramic membrane and the amount of high-cost dopant.

#### 2.3.1. Li and La Sublattice

The partial substitution of Li^+^ and La^3+^ ions in the LLZ structure was studied in works [99,100,101,102]. Sodhiya et al. [99] used Al^3+^ and Ba^2+^ for co-doping LLZ on Li and La sublattices–Li_6.28+x_Al_0.24_La_3−x_Ba_x_Zr_2_O_12_ (x = 0.0–0.3). Li_6.38_Al_0.24_La_2.9_Ba_0.1_Zr_2_O_12_ annealed at 1000 °C had the cubic structure, but a low conductivity value—4.6 × 10^−5^ S/cm at 25 °C. Li^+^ substitution by Ga^3+^ and La^3+^ by divalent and trivalent ions was presented in several works [100,101,102]. Li et al. [100] studied the effect of Ga^3+^ and Yb^3+^ co-doping on the structure and ionic conductivity of LLZ—Li_6.4_Ga_0.2_La_3−x_Yb_x_Zr_2_O_12_ (x = 0–0.1). The solid electrolyte with x = 0.05 showed the highest ionic conductivity of 8.96 × 10^−4^ S/cm, which was attributed to the optimized Li^+^ transport channels and ceramics densification. Shen et al. [101] synthesized Ga and Sr co-doped LLZ—Li_6.4+x_Ga_0.2_La_3−x_Sr_x_Zr_2_O_12_ (x = 0–0.4). Sr^2+^ ions were used to enhance the Li^+^ concentration, and their introduction led to conductivity growth up to 5.5 × 10^−4^ S/cm for Li_6.5_Ga_0.2_La_2.9_Sr_0.1_Zr_2_O_12_. Luo et al. [102] obtained Ga and Y co-doped LLZ—Li_7−3x_Ga_x_La_3−y_Y_y_Zr_2_O_12_ (x = 0.2 and y = 0–0.5). The solid electrolyte with y = 0.25 had the highest total conductivity (1.61 × 10^−3^ S/cm at room temperature) and relative density (96.6%). Therefore, the positive effect of co-doping of Y-doped LLZ (part 2.2.2) by Ga^3+^ can be observed.

#### 2.3.2. Li and Zr Sublattice

The simultaneous substitution in Li and Zr sublattices is the most widely represented and even wider in reality because of the uncontrolled introduction of aluminum from crucibles [20,28,56,60,61,73,80,81,88]. The attempt of LLZ dual-doping using Ta^5+^ and Al^3+^ or Ta^5+^ and Ga^3+^ was made by Allen et al. [103], and the following compositions were obtained: Li_6.15_La_3_Zr_1.75_Ta_0.25_Al_0.2_O_12_ and Li_6.15_La_3_Zr_1.75_Ta_0.25_Ga_0.2_O_12_. However, the addition of Al or Ga to Ta-doped LLZ led to the conductivity decrease from 0.87 mS/cm to 0.37 or 0.41 mS/cm, respectively. In our previous work [104], the addition of Al to Ta-doped LLZ also did not lead to significant conductivity growth; it changed from 1.1 × 10^−4^ (Li_6.4_La_3_Zr_1.4_Ta_0.6_O_12_) to 2.0 × 10^−4^ S/cm (Li_6.3_Al_0.05_La_3_Zr_1.6_Ta_0.6_O_12_) at 20 °C. However, according to DRT data, the Ta co-doping of Al-doped LLZ, as well as increasing Li vacancy amount leads to the formation of a more open space for Li^+^ transport due to some Al movement from 24d to 96h Li sites [96]. High values of total conductivity with the substitution of Li^+^ by Al^3+^ and Zr^4+^ by Ta^5+^ were achieved in several works [105,106,107]. Xue et al. [105] used such a co-doping strategy; the ionic conductivity improvement and reduction of Ta were achieved. The ionic conductivity of Li_6.25_La_3_Zr_1.55_Al_0.1_Ta_0.45_O_12_ solid electrolyte was equal to 6.7 × 10^−4^ S/cm at 25 °C. Matsuda et al. [106] obtained Li_6.6−z/2_Al_z/2_La_3_Zr_1.6+z_Ta_0.4−z_O_12_ solid electrolytes; the highest value of total conductivity was reached for ceramic with z = 0.275—1.03 × 10^−3^ S/cm at 25 °C. Yan et al. [107] obtained Li_5.85_Al_0.25_La_3_Zr_1.6_Ta_0.4_O_12_ solid electrolyte with ionic conductivity of 4.59 × 10^−4^ S/cm at 20 °C. Song [98] used the substitution of Li^+^ by Ga^3+^and Zr^4+^ by Ta^5+^/Ba^2+^; the highest ionic conductivity of 1.02 × 10^−3^ S/cm at room temperature was achieved for Li_6.4_Ga_0.1_La_3_Zr_1.55_Ba_0.05_Ta_0.4_O_12_ ceramic. In our previous work [108], the solid electrolytes with the partial substitution of Li^+^ by Al^3+^ and Zr^4+^ by Nb^5+^ in LLZ (Li_6.75−3x_Al_x_La_3_Zr_1.75_Nb_0.25_O_12_) were synthesized using the sol–gel method. The introduction of Al (x = 0.05) into Nb-doped LLZ led to room-temperature conductivity growth from 4.0 × 10^−5^ to 6.3 × 10^−4^ S/cm.

Other elements were also used for the co-doping of Al-doped and Ga-doped LLZ. For example, Li et al. [109] obtained solid electrolytes with the substitution of Zr^4+^ by Mo^6+^ in Al-doped LLZ. The total conductivity of Li_6.375_Al_0.075_La_3_Zr_1.8_Mo_0.2_O_12_ sintered at 1040 °C achieved 4.41 × 10^−4^ S/cm. Wu et al. [110] co-doped LLZ with Al^3+^ and Ti^4+^; room-temperature total conductivity was equal to 1.51 × 10^−4^ S/cm for Li_6.25_Al_0.25_La_3_Zr_1.75_Ti_0.25_O_12_ solid electrolyte annealed at 900 °C. Yang et al. [111] synthesized Li_6.925−3x_Al_x_La_3_Zr_1.925_Sb_0.075_O_12_ solid electrolytes with different Al content. It was established that such dual substitution led to the densification and ionic conductivity improvement of solid electrolytes. Li_6.775_Al_0.05_La_3_Zr_1.925_Sb_0.075_O_12_ exhibited the highest total conductivity, 4.1 × 10^−4^ S/cm at 30 °C. Alizadeh et al. [112] used the dual-doping of LLZ by Ga^3+^ and Y^3+^ (Li_6.4_Ga_0.2_La_3_Zr_2−x_Y_x_O_12_). The Li_6.4_Ga_0.2_La_3_Zr_21.7_Y_0.3_O_12_ solid electrolyte had the maximum ionic conductivity (1.04 × 10^−3^ S/cm 25 °C); such high values of conductivity can be caused by the efficiency of the Ga/Y introduction to promote ceramics sintering. Simultaneously with Li^+^ substitution by Ga^3+^, Zr^4+^ was partially substituted by Sc^3+^ to increase Li^+^ concentration in the work of Buannic et al. [113] and Li_6.65_Ga_0.15_La_3_Zr_1.90_Sc_0.10_O_12_ solid electrolyte with lithium-ion conductivity of 1.8 × 10^−3^ S/cm at 27 °C was obtained.

#### 2.3.3. La and Zr Sublattice

In several works, the co-doping of Ta-doped LLZ on an La sublattice by A^2+^/A^3+^ is presented [94,97,114,115,116,117]. Wang et al. [114] synthesized Ta-doped LLZ with the partial substitution of La^3+^ by Ba^2+^ with a larger ion radius; such substitution was used to enlarge channels for Li^+^ transport. Li_6.46_La_2.94_Ba_0.06_Zr_1.4_Ta_0.6_O_12_ exhibited the highest conductivity—6.04 × 10^−4^ S/cm. Dhivya and Murugan [115] studied the effect of the simultaneous substitution of La^3+^ by Y^3+^ and Zr^4+^ by Ta^5+^ in the LLZ structure. Such structure modifications led to increasing grain-boundary and bulk conductivities of the samples; Li_6.6_La_2.75_Y_0.25_Zr_1.6_Ta_0.4_O_12_ exhibited the highest room-temperature conductivity—4.36 × 10^−4^ S/cm. Zhang et al. [116] studied the effect of Ca^2+^ and Ta^5+^ co-doping on the conductivity of LLZ. Li_7_La_3−x_Ca_x_Zr_2−y_Ta_y_O_12_ with x = y = 0.25 had the highest value of conductivity—7.65 × 10^−4^ S/cm at room temperature. Chen et al. [117] also used Ca^2+^ and Ta^5+^co-doping for the improvement of LLZ lithium-ion conductivity. Maximum conductivity was also observed after the small addition of Ca (0.05) to Li_6.4_La_2.95_Ta_0.6_Zr_1.4_O_12_ solid electrolyte, 2.84 × 10^−4^ S/cm.

**Figure 7:**
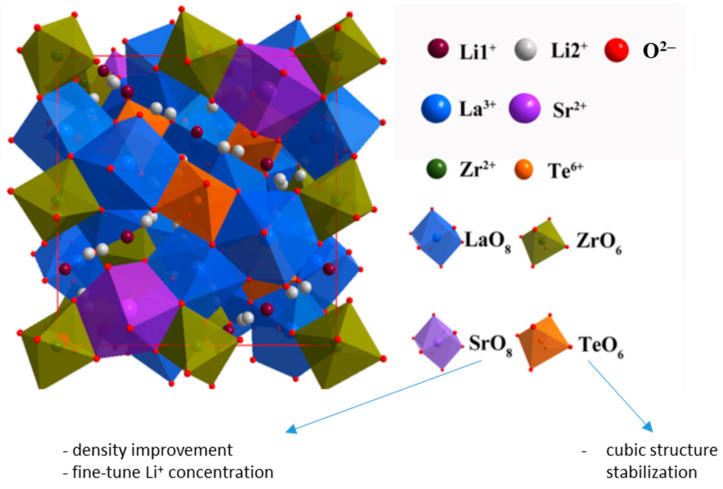
The crystal structure of Li_6.3+x_La_3−x_Sr_x_Zr_1.65_Te_0.35_O_12_ (x = 0.125–0.25) electrolytes [120]. Reprinted from [120], with permission from Elsevier B.V.

Cao et al. [118] synthesized solid electrolytes based on LLZ with dual-doping by Sb^5+^ and Y^3+^ on Zr^4+^ and La^3+^ sites. The Li_6.925_La_2.95_Y_0.05_Zr_1.925_Sb_0.075_O_12_ solid electrolyte showed the highest value of total conductivity at room temperature (3.2 × 10^−4^ S/cm). In Ref. [119], the simultaneous substitution of La^3+^ by Ba^2+^ and Zr^4+^ by Sb^5+^ was used; the total conductivity of Li_6.945_La_2.98_Ba_0.02_Zr_1.925_Sb_0.075_O_12_ solid electrolyte was equal to 1.53 × 10^−4^ S/cm. Shen et al. [120] obtained solid electrolytes based on LLZ with Sr^2+^ and Te^6+^ co-doping, Figure 7. Te-doping was used for cubic structure stabilization, and Sr^2+^ co-doping was carried out to improve electrolyte density and grain contact, as well as to achieve suitable Li^+^ concentration and migration channel size. It was established that additional substitution of a compound by strontium led to the conductivity growth (4.27 × 10^−4^ S/cm for Li_6.425_La_2.875_Sr_0.125_Zr_1.65_Te_0.35_O_12_). Moreover, the obtained solid electrolyte showed good cycle stability in symmetric cells with Li.

- It can be concluded that solid electrolytes with the simultaneous substitution of La^3+^ and Zr^4+^ in the LLZ structure have lower conductivity values in comparison with Li/Zr and Li/La substituted garnets. Al and Ga are used as co-doping elements for the simultaneous substitution in Li/Zr and Li/La sublattices in the LLZ structure (Section 2.3.1 and Section 2.3.2) for lithium-ion conductivity improvement of solid electrolytes. As mentioned above (Section 2.2.1), these dopants act as sintering additives, which leads to ceramic densification and grain-boundary conductivity improvement.

#### 2.3.4. Li, Zr, and La Sublattice

Simultaneous doping on the three sublattices in the LLZ structure has also been studied [97,121,122,123]. Meesala et al. [97] synthesized the ternary-substituted Li_6.65_Ga_0.05_La_2.95_Ba_0.05_Zr_1.75_Ta_0.25_O_12_ solid electrolyte with a cubic structure. The ternary-substitution led to the conductivity growth up to 7.2 × 10^−4^ S/cm. Liu et al. [121] studied the effect of Ba^2+^ and Y^3+^ or Ba^2+^ and W^6+^ co-doping on the conductivity of Al-doped LLZ. The lithium-ion conductivities of Li_6.52_Al_0.2_La_2.98_Ba_0.02_Zr_1.9_Y_0.1_O_12_ and Li_5.72_Al_0.2_La_2.98_Ba_0.02_Zr_1.65_W_0.35_O_12_ solid electrolytes are equal to 2.02 × 10^−4^ and 5.35 × 10^−4^ S/cm, respectively. Li_6.52_Al_0.2_La_2.98_Ba_0.02_Zr_1.9_Y_0.1_O_12_ solid electrolyte synthesized in work [122] exhibited room-temperature lithium-ion conductivity of 2.96 × 10^−4^ S/cm. Limpert et al. [123] studied the influence of lithium content in Li_6.75±x_La_2.75_Ca_0.25_Zr_1.75_Nb_0.5_O_12_ on the total conductivity of the ceramics. The sintering was carried out in Al_2_O_3_ and MgO crucibles. It was shown that samples annealed in Al_2_O_3_ crucibles had higher values of conductivity due to aluminum penetration; the maximum conductivity (1.68 × 10^−4^ S/cm) was achieved for a solid electrolyte with lithium content equal to 6.37 mol. 

The compositions of Li_7_La_3_Zr_2_O_12_ solid electrolytes with dual- and multi-doping on several sublattices and their total conductivity values are presented in Table 1. It can be seen that compounds with partial substitution of Li^+^ and Zr^4+^ in the LLZ structure possess higher lithium-ion conductivity values. Simultaneous doping on Li, La, and Zr sites did not lead to the significant conductivity improvement of ceramic electrolytes. Moreover, it should be noted that lithium-ion conductivity improvement was successfully achieved for dual-doped LLZ with the partial substitution of Li^+^ by Ga^3+^.

### 2.4. Mono- and Multi-Doping: Comparison and Trends

**Figure 8:**
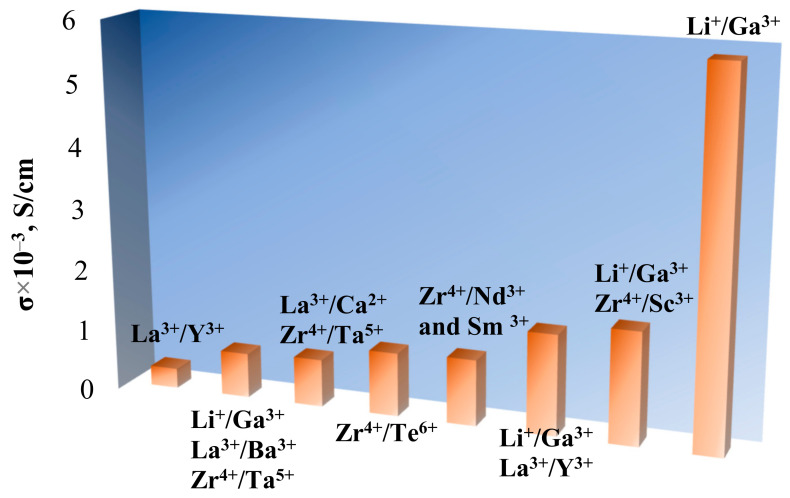
Total conductivity of solid electrolytes with mono-, dual-, and multi-doping of Li_7_La_3_Zr_2_O_12_ on different sublattices.

The cubic phase of Li_7_La_3_Zr_2_O_12_ can be successfully stabilized by various chemical elements. The higher conductivity values of Li_7_La_3_Zr_2_O_12_ solid electrolytes with mono-, dual-, and multi-doping on different sublattices are shown in Figure 8. It can be concluded that:

- solid electrolytes with mono-, dual-, and multi-doping possess the highest Li-ion conductivity if Li^+^ was partially substituted by Ga^3+^;
- the highest values were achieved for a solid electrolyte with the partial substitution of Li^+^ by Ga^3+^ synthesized using the sol–gel method. Hot-pressing and spark plasma sintering techniques are also effective methods for obtaining high-density ceramics.

**Figure 9:**
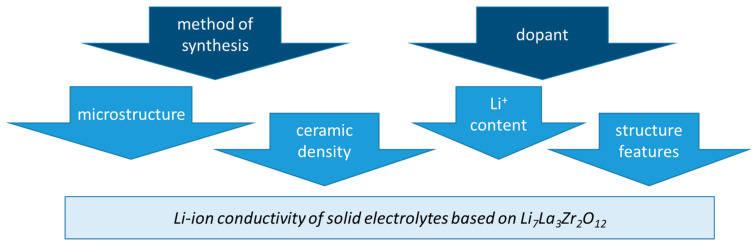
Factors influencing the lithium-ion conductivity of the solid electrolytes based on Li_7_La_3_Zr_2_O_12_.

Figure 9 summarizes the factors that influence the lithium-ion conductivity of the solid electrolytes based on Li_7_La_3_Zr_2_O_12_. It should be taken into account that not only the choice of dopant elements and their number influence Li^+^-conductivity but also the method of ceramic electrolyte synthesis. These two factors determine the garnet structure (Li occupancy at the tetrahedral and octahedral sites) and Li^+^ content in the solid electrolyte, its density, and microstructure features.

Thus, the dual-doping strategy with simultaneous substitution in Li and Zr or Li and La sublattices by Al or Ga co-doping elements is one of the most effective techniques for conductivity, density, and stability improvement of solid electrolytes based on LLZ. Modification of the synthesis method can also significantly improve sample densification and microstructure: (1) replacement of the traditional solid-phase synthesis to the sol–gel method; (2) use of hot-pressing or spark plasma sintering technique. It should be noted that solid electrolytes based on LLZ with different dopants have good chemical compatibility with most widely used electrode materials (LiCoO_2_, LiNi_1/3_Co_1/3_Mn_1/3_O_2_, LiFePO_4_, Li_4_Ti_5_O_12_, and Li) [20].

The next important step for the application of solid electrolytes based on Li_7_La_3_Zr_2_O_12_ with improved lithium-ion conductivity in all-solid-state batteries is the transition from bulk samples to thin films because the thickness of the solid electrolyte has a significant impact on the electrochemical characteristics of ASSB. Thin films of doped LLZ were obtained using different techniques: spin or dip coating, atomic layer deposition, pulsed laser deposition, radio frequency magnetron sputtering, and tape casting [124]. However, researchers are faced with underestimated values of lithium-ion conductivity of LLZ thin films in comparison with bulk analogs. Among the presented in the literature methods of film formation, the tape-casting approach allows the fabrication of thin LLZ ceramic on a large scale. To increase the density of the formed films, some sintering additives can be used [125].

## 3. Conclusions

Li_7_La_3_Zr_2_O_12_ with a high-conductive cubic structure is one of the most promising solid electrolytes for lithium and lithium-ion all-solid-state batteries. A large number of studies have been devoted to the stabilization of highly conductive LLZ structures using a doping strategy. The total lithium-ion conductivity of solid electrolytes based on Li_7_La_3_Zr_2_O_12_ is caused by a set of factors: bulk conductivity depends on garnet framework features (Li content, Li site occupancies, Li^+^ transport channels), while grain-boundary conductivity depends on microstructure, density of ceramic membrane, and the presence of impurities. 

The lithium-ion conductivity of LLZ ceramics greatly depends on the conditions of its synthesis and heat treatment, which affects sample densification and lithium content. Therefore, it is necessary not only to choose the doping strategy but also to choose the optimal synthesis method for obtaining highly conductive solid electrolytes based on Li_7_La_3_Zr_2_O_12_. According to the literature data, traditional solid-phase synthesis is more often used to obtain doped LLZ. However, target properties of solid electrolytes can be improved by the application of hot-pressing or spark plasma sintering techniques, or using the sol–gel method. 

High values of lithium-ion conductivity were achieved by partial substitution in different sublattices of Li_7_La_3_Zr_2_O_12_. Al^3+^ and Ga^3+^ doping on the Li^+^ site led to cubic modification stabilization and lithium-ion conductivity improvement (up to 7.81 mS/cm at 30 °C for Ga-doped LLZ) because of Li^+^ vacancy formation and samples densification, while the low conductivity of doped LLZ may be caused by the poor density of ceramics or impurity phase formation. The strategy of dual- and multi-doping LLZ was proposed for structuring a better Li^+^ conduction framework in LLZ (Li occupancy at the tetrahedral and octahedral sites), the improvement of membrane densification and air stability; moreover, in some cases, it can reduce the amount of high-cost dopant. Simultaneous substitution of Li^+^ and Zr^4+^ ions in the LLZ structure is the most widely represented. The highest lithium-ion conductivity values of the solid electrolytes with double doping were achieved for the partial substitution of Li^+^ by Ga^3+^ in the LLZ structure. Simultaneous doping on Li, La, and Zr sites did not lead to the significant conductivity improvement of ceramic membranes. In addition, it also should be taken into account that the chosen dopant should not have a negative effect on the lithium-ion conductivity unipolarity of the solid electrolyte and its stability to electrode materials, including lithium metal. It can be concluded that the doping strategies presented in the literature proved to be effective at improving the conductivity and density of solid electrolytes based on Li_7_La_3_Zr_2_O_12_.

The obtained solid electrolytes with a garnet structure can be used in different electrochemical devices: lithium or lithium-ion batteries (including all-solid-state batteries), lithium-air, and aqueous rechargeable batteries. However, despite the good compatibility of LLZ-based electrolytes with most commercially available electrode materials, as well as with the developing technologies for obtaining thin-film ceramic membranes, some challenges need to be addressed before Li_7_La_3_Zr_2_O_12_ can be used in commercial devices:

- lower conductivity values of thin-film electrolytes based on LLZ compared to bulk ceramics (10^−3^ S/cm at room temperature);
- the problem of contact between the cathode and solid electrolyte (high resistance at the interface) is still the most acute problem for the successful application of solid electrolytes.

Therefore, future research should be focused on the development of new approaches to obtain high-quality Li_7_La_3_Zr_2_O_12_ films with improved ionic conductivity and to optimize electrode–electrolyte interface.

## Figures and Tables

**Table 1:** Total conductivity of multi-doped Li_7_La_3_Zr_2_O_12_ solid electrolytes prepared by different methods.

Solid Electrolyte	Room-Temperature Total Conductivity, S/cm	Synthesis Method	Reference
Li and La sublattice			
Li_6.38_Al_0.24_La_2.9_Ba_0.1_Zr_2_O_12_	4.6 × 10^−5^	solid-state reaction	[99]
Li_6.4_Ga_0.2_La_2.95_Yb_0.05_Zr_2_O_12_	8.96 × 10^−4^	solid-state reaction	[100]
Li_6.5_Ga_0.2_La_2.9_Sr_0.1_Zr_2_O_12_	5.5 × 10^−4^	solid-state reaction	[101]
Li_6.4_Ga_0.2_La_2.75_Y_0.25_Zr_2_O_12_	1.61 × 10^−3^	solid-state reaction	[102]
Li and Zr sublattice			
Li_6.15_La_3_Zr_1.75_Ta_0.25_Ga_0.2_O_12_	4.1 × 10^−4^	co-precipitated method	[103]
Li_6.15_La_3_Zr_1.75_Ta_0.25_Al_0.2_O_12_	3.7 × 10^−4^	co-precipitated method	[103]
Li_6.3_Al_0.05_La_3_Zr_1.6_Ta_0.6_O_12_	2.0 × 10^−4^	sol–gel method	[104]
Li_6.25_La_3_Zr_1.55_Al_0.1_Ta_0.45_O_12_	6.7 × 10^−4^	sol–gel method	[105]
Li_6.4625_Al_0.1375_La_3_Zr_1.875_Ta_0.125_O_12_	1.03 × 10^−3^	solid-state reaction	[106]
Li_5.85_Al_0.25_La_3_Zr_1.6_Ta_0.4_O_12_	4.59 × 10^−4^	solid-state reaction	[107]
Li_6.4_Ga_0.1_La_3_Zr_1.55_Ba_0.05_Ta_0.4_O_12_	1.02 × 10^−3^	solid-state reaction	[98]
Li_6.6_Al_0.05_La_3_Zr_1.75_Nb_0.25_O_12_	6.3 × 10^−4^	sol–gel method	[108]
Li_6.375_Al_0.075_La_3_Zr_1.8_Mo_0.2_O_12_	4.41 × 10^−4^	sol–gel method	[109]
Li_6.25_Al_0.25_La_3_Zr_1.75_Ti_0.25_O_12_	1.51 × 10^−4^	sol–gel method	[110]
Li_6.775_Al_0.05_La_3_Zr_1.925_Sb_0.075_O_12_	4.1 × 10^−4^	solid-state reaction	[111]
Li_6.4_Ga_0.2_La_3_Zr_21.7_Y_0.3_O_12_	1.04 × 10^−3^	sol–gel method	[112]
Li_6.65_Ga_0.15_La_3_Zr_1.90_Sc_0.10_O_12_	1.8 × 10^−3^	sol–gel method	[113]
La and Zr sublattice			
Li_6.8_La_2.95_Ba_0.05_Zr_1.75_Ta_0.25_O_12_	6.5 × 10^−4^	solid-state reaction	[97]
Li_6.46_La_2.94_Ba_0.06_Zr_1.4_Ta_0.6_O_12_	6.04 × 10^−4^	solid-state reaction	[114]
Li_6.6_La_2.75_Y_0.25_Zr_1.6_Ta_0.4_O_12_	4.36 × 10^−4^	solid-state reaction	[115]
Li_7_La_2.75_Ca_0.25_Zr_1.75_Ta_0.25_O_12_	7.65 × 10^−4^	solid-state reaction	[116]
Li_6.4_La_2.95_Ca_0.05_Ta_0.6_Zr_1.4_O_12_	2.84 × 10^−4^	solution method	[117]
Li_6.925_La_2.95_Y_0.05_Zr_1.925_Sb_0.075_O_12_	3.2 × 10^−4^	solid-state reaction	[118]
Li_6.945_La_2.98_Ba_0.02_Zr_1.925_Sb_0.075_O_12_	1.53 × 10^−4^	solid-state reaction	[119]
Li_6.425_La_2.875_Sr_0.125_Zr_1.65_Te_0.35_O_12_	4.27 × 10^−4^	solid-state reaction	[120]
Li, Zr and La sublattice			
Li_6.65_Ga_0.05_La_2.95_Ba_0.05_Zr_1.75_Ta_0.25_O_12_	7.2 × 10^−4^	solid-state reaction	[97]
Li_6.52_Al_0.2_La_2.98_Ba_0.02_Zr_1.9_Y_0.1_O_12_	2.02 × 10^−4^	solid-state reaction	[121]
Li_5.72_Al_0.2_La_2.98_Ba_0.02_Zr_1.65_W_0.35_O_12_	5.35 × 10^−4^	solid-state reaction	[121]
Li_6.52_Al_0.2_La_2.98_Ba_0.02_Zr_1.9_Y_0.1_O_12_	2.96 × 10^−4^	solid-state reaction	[122]
Li_6.75±x_La_2.75_Ca_0.25_Zr_1.75_Nb_0.5_O_12_	1.68 × 10^−4^	solid-state reaction	[123]

## Data Availability

The data presented in this study are available from the corresponding author upon request.
